# Implications of TORCH Diseases in Retinal Development—Special Focus on Congenital Toxoplasmosis

**DOI:** 10.3389/fcimb.2020.585727

**Published:** 2020-10-26

**Authors:** Viviane Souza de Campos, Karin C. Calaza, Daniel Adesse

**Affiliations:** ^1^Laboratório de Neurobiologia da Retina, Instituto de Biologia, Universidade Federal Fluminense, Niteroi, Brazil; ^2^Laboratório de Biologia Estrutural, Instituto Oswaldo Cruz, Fiocruz, Rio de Janeiro, Brazil

**Keywords:** congenital toxoplasmosis, TORCH, retinal development, *Toxoplasma gondii*, congenital infections, teratogenesis

## Abstract

There are certain critical periods during pregnancy when the fetus is at high risk for exposure to teratogens. Some microorganisms, including *Toxoplasma gondii*, are known to exhibit teratogenic effects, interfering with fetal development and causing irreversible disturbances. *T. gondii* is an obligate intracellular parasite and the etiological agent of Toxoplasmosis, a zoonosis that affects one third of the world's population. Although congenital infection can cause severe fetal damage, the injury extension depends on the gestational period of infection, among other factors, like parasite genotype and host immunity. This parasite invades the Central Nervous System (CNS), forming tissue cysts, and can interfere with neurodevelopment, leading to frequent neurological abnormalities associated with *T. gondii* infection. Therefore, *T. gondii* is included in the **TORCH** complex of infectious diseases that may lead to neurological malformations (**T**oxoplasmosis, **O**thers, **R**ubella, **C**ytomegalovirus, and **H**erpes). The retina is part of CNS, as it is derived from the diencephalon. Except for astrocytes and microglia, retinal cells originate from multipotent neural progenitors. After cell cycle exit, cells migrate to specific layers, undergo morphological and neurochemical differentiation, form synapses and establish their circuits. The retina is organized in nuclear layers intercalated by plexus, responsible for translating and preprocessing light stimuli and for sending this information to the brain visual nuclei for image perception. Ocular toxoplasmosis (OT) is a very debilitating condition and may present high severity in areas in which virulent strains are found. However, little is known about the effect of congenital infection on the biology of retinal progenitors/ immature cells and how this infection may affect the development of this tissue. In this context, this study reviews the effects that congenital infections may cause to the developing retina and the cellular and molecular aspects of these diseases, with special focus on congenital OT.

## The Retina and Vision

For many vertebrates, especially humans, the main environmental perception mechanism is the sense of vision. Vision determines physiological behaviors such as feeding, predation, and in the case of humans, complex social behaviors, such as bonding and the ability to recognize people's emotions by observing their faces. This fundamental skill is permitted by the presence of the visual system. Visual impairment can limit people's ability to perform everyday tasks, and impaired interaction with the surrounding world affects quality of life. People with visual impairment are three times more likely to suffer from depression and anxiety disorders and to be unemployed. Therefore, it is particularly important to prevent and/or to treat visual impairment and research therapeutic alternatives to vision pathologies. The tissue responsible for the transduction of light stimulus and pre-processing of visual information is the retina, a highly organized network of nerve cells located in the back of the eye.

**Figure 1:**
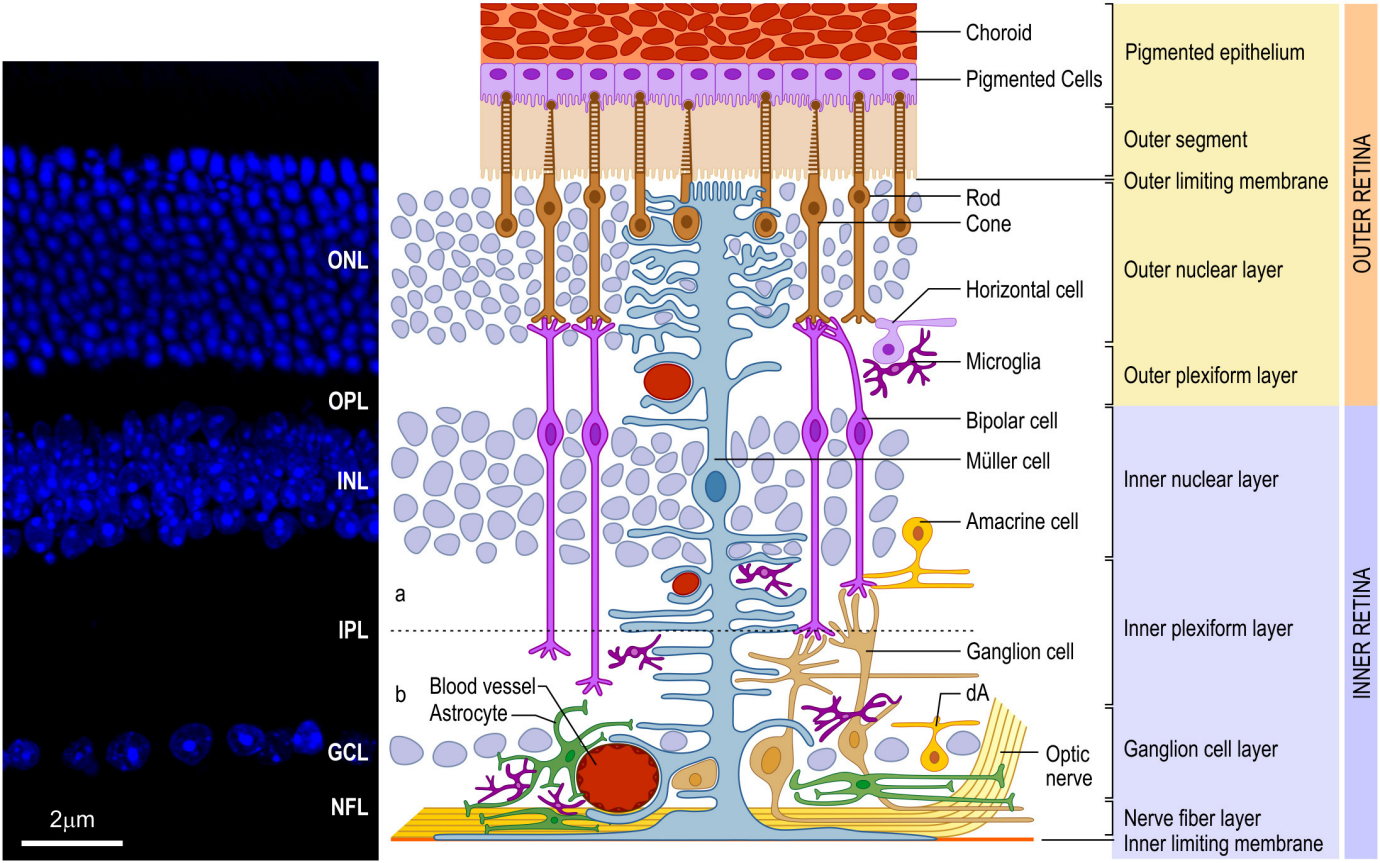
Schematic organization of the vertebrate retina. **(Left)** Vertical section of mouse retina labeled with nuclear marker DAPI revealing the organization of the retina in layers of cell bodies and process named as follows: ganglion cell layer (GCL), inner plexiform layer (IPL), inner nuclear layer (INL), outer plexiform layer (OPL), outer nuclear layer (ONL), and inner/outer segment of photoreceptors (IS/OS). **(Right)** The retina consists of different cell types located in specific layers. Cell bodies of rod and cone photoreceptors (brown) are located in the ONL. Both photoreceptors perform synapses in the OPL with bipolar (purple) and horizontal (lilac) cells, which, in turn, show the cell bodies in the outer portion of INL, as well as amacrine (yellow) cells and Müller (blue) cells. Bipolar and amacrine cells arborize in the IPL, contacting ganglion cell dendrites. Rod bipolar cells arborizes in the inner portion of IPL. Cone bipolar cells (right pair of purple cells) are subdivided in ON and OFF bipolar cell contacting the same cone in the OPL and synapsing, respectively, in the ON (b) or OFF portion of the IPL [dashed line shows the functional division of the IPL in OFF (a) and ON (b) circuitries. Ganglion (orange), as well as displaced amacrine (dA) cell bodies are situated in the GCL. Axons of ganglion cells form the nerve fiber layer (NFL), leave the retina through the optic head nerve taking the information to visual brain nuclei by optic nerve (ON). The three types of glial cells (Müller, astrocytes, and microglia) are found in different retinal layers. Astrocytes (green) are restricted to the inner portion of the retina, in the NFL and GCL, and have a close relation to blood vessels. Microglia (dark purple) appear preferentially in the plexiform layers (IPL and OPL). Finally, Müller cells, the predominant glia in the retina, extend their processes radially throughout the retina forming the inner limiting membrane (ILM) and the outer limiting membrane (OLM). Muller glia processes interacts with almost all retinal cell types, including blood vessels, displaying a crucial role in the physiology of this tissue. In mice and humans, the inner retina is vascularized by three capillary branches of central retinal artery. The outer retina, with the avascular photoreceptor region, relies on the choriocapillaris (Ch) lying beneath the retinal pigment epithelium (RPE)]. Scale bar: 20 μm.

The vertebrate retina presents a well-conserved laminar organization with three layers of cell bodies, intercalated by two layers of synaptic contacts (Figure 1). The outer nuclear layer (ONL) lies in the outer portion of the retina, close to the choroid, and contains the photoreceptor cell bodies (cones and rods). The inner nuclear layer (INL), in turn, contains the cell bodies of horizontal, bipolar, amacrine and Müller glia, and a small number of interplexiform cells and displaced ganglion cells. Finally, in the inner portion of the retina, closer to the vitreous chamber of the eye, lies the ganglion cell layer (GCL), which comprises the cell bodies of retinal ganglion cells (RGCs) and displaced amacrine cells. All these neuronal cell types communicate through synapses, forming the outer (OPL) and inner (IPL) plexiform layers. In the innermost portion of the retina, axons from the RGCs form the nerve fiber layer (NFL). These axons transmit the preprocessed information from the retina to the brain visual nuclei through the optic nerve (ON). It is important to note that, contiguous to the photoreceptors, located in the outermost part of the retina, lies the retinal pigmented epithelium (RPE), an important player for appropriate retinal development and for the physiology of the mature retina (for review, Strauss, 2005).

Photoreceptors contain photosensitive molecules (visual pigments) that enable the transduction of the light stimulus. These visual pigments are located in the outer portion of photoreceptors, named the outer segment. Rods and cones possess a specific visual pigment and, due to morphological and neurochemical specializations, work under different light conditions. As rods are highly sensitive to light, they mediate vision in dim light (scotopic) conditions, such as during the night. Although cones are less sensitive to light, mediating vision in daylight conditions (photopic), the cone circuitry is responsible for the high resolution (temporal and spatial) capacity of the retina and for color vision (Kolb, 2003).

Bipolar cells receive information from photoreceptors through synapses in the OPL, transferring it to ganglion cells, in IPL synapses, forming the radial pathway of the retina. Horizontal and amacrine cells modulate this signal through the horizontal/lateral pathway, enriching retina performance (visual acuity, contrast, among others).

Regarding the glial cells present in the mature retina, the main cell types are Müller glia, astrocytes and microglia. Müller glia is the only type of glia originated from retinal precursors (Turner and Cepko, 1987) and the predominant retinal glia in all species. Its cell body is located in the middle of the INL, with processes extending throughout the entire radial thickness of the retina, which arborize to form the outer (OLM) and inner (ILM) limiting membranes (Figure 1) (Bringmann and Reichenbach, 2001; Newman, 2004). Astrocytes, non-retinal originated cells, migrate to the retina through the optic nerve (ON) during development (Stone and Dreher, 1987; Chan-Ling, 1994), and assume a location in the NFL (Figure 1) where they establish a close relationship with blood capillaries. The microglia, a mesodermal originated cell, also migrates to the retina during development (Chan-Ling, 1994) forming a resident population located mainly in the OPL, IPL and NFL (Figure 1).

The blood supply of the mammalian retina comes from two sources, the central retinal artery (CRA) and from choroidal vascularization (Figure 1). In humans, the CRA is derived from the ophthalmic artery, a branch of the internal carotid artery. It enters the eye through the optic disc and branches into the retina, forming peripapillary and intraretinal (inner and outer) beds, which supply blood to the innermost layers of the retina. The peripapillary bed is located in the innermost portion of the NFL, while the inner intraretinal bed is located in the GCL, and the outer intraretinal bed occupies the IPL and INL to the OPL. Mouse retina follows the same vascular organization as the human retina but, interestingly, in the rat retina, the peripapillary bed is absent. The outermost layers of the retina, especially the ONL, depend on the choroidal vascularization supply (Zhang, 1994).

## Retinal Development

The retina is a highly organized tissue with a complex array of synapses resulting in efficient light information transduction, pre-processing, and transmission. Therefore, retinal development must produce the right number of different retinal cell types, as well as the functional circuitries. According to this, this phenomenon is highly regulated by both intrinsic and extrinsic factors.

The retina originates from the posterior part of the forebrain, the diencephalon (Hamburger and Hamilton, 1951; Ambroise-Thomas and Petersen, 2000), being a part of the central nervous system. Optical vesicles generated from the diencephalon undergo invaginations to form the optic cup (Smirnov and Puchkov, 2004; Heavner and Pevny, 2012). The inner wall of this structure forms the retina, while the outer wall forms the pigmented epithelium (Ambroise-Thomas and Petersen, 2000; Smirnov and Puchkov, 2004; Fan et al., 2016).

Early vertebrate retinogenesis is characterized by two aspects: the multipotency of retinal progenitor cells (RPC) and the well-conserved birth order of retinal cell subtypes (Dyer and Cepko, 2001; Marquardt and Gruss, 2002). In particular, retinal progenitor subpopulations have the ability to originate all neurons found in the retina, demonstrating the multipotent nature of these cells (Wetts and Fraser, 1988). Concerning the conserved order of retinal cell birth, ganglion cells are the first cells to be generated while bipolar cells are typically the last (Carter-Dawson and Lavail, 1979; Dräger, 1985; Young, 1985; Prada et al., 1991, Figure 2). However, the generation period of a given cell type usually overlaps and correlates with that of another cell type during the embryonic and/or postnatal period, depending on the species (Young, 1985; Prada et al., 1991; Cepko et al., 1996; Georges et al., 1999; Yang, 2004; Voinescu et al., 2009). It is noteworthy that all retinal development phenomena (cell generation, programmed cell death and synaptogenesis, among others) occur in a central-periphery gradient, with central regions (fovea in the human retina) maturing first, followed by the periphery.

**Figure 2:**
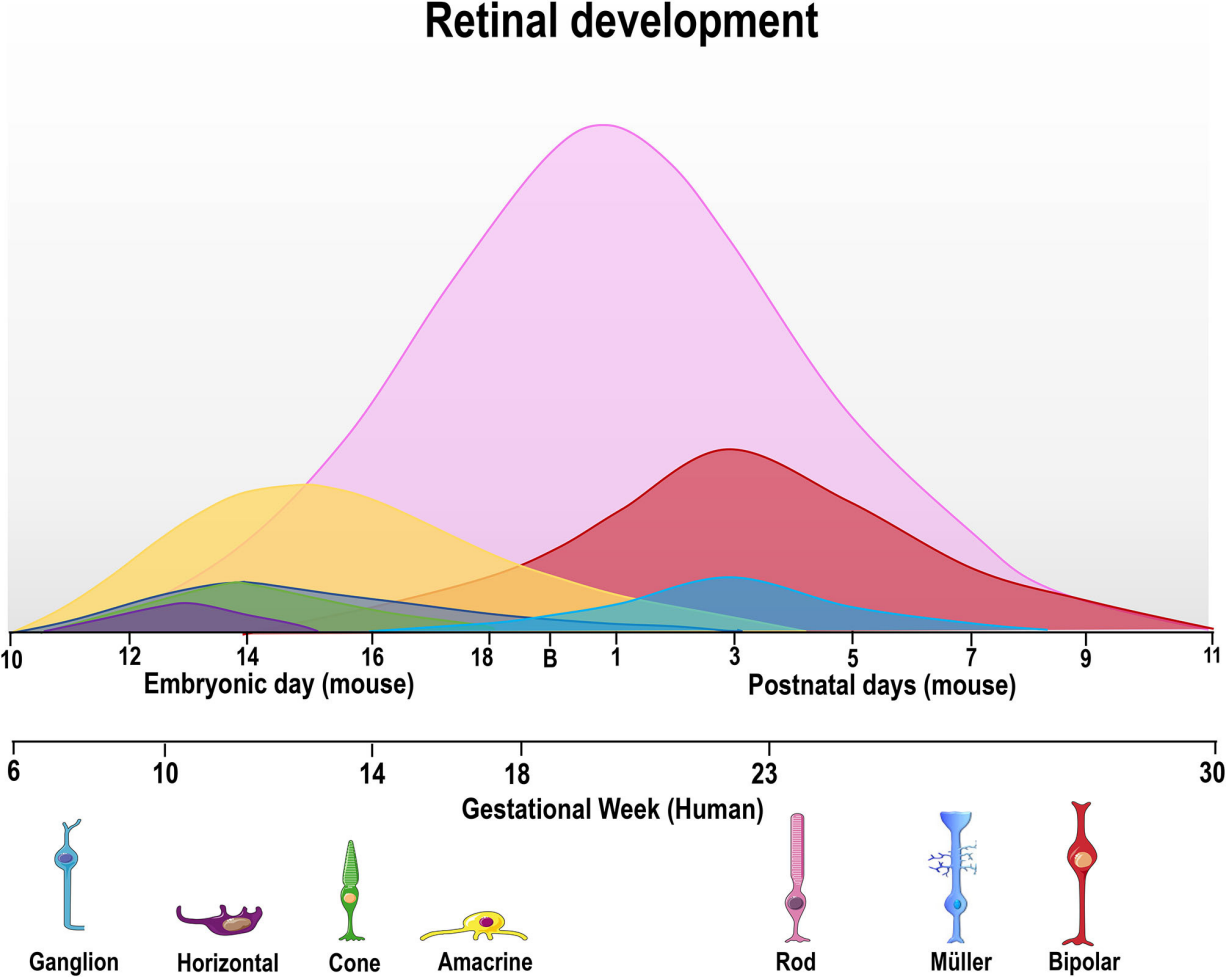
Time course of retinal neurogenesis in human and mice. There are two main waves of retinal cell birth from early and late progenitors. The first wave begins around embryonic day (E) 10–18 in mice and gestational week (GW) 6–18 in humans, with ganglion (dark blue) cells being the first cell type to exit the cell cycle, followed by horizontal (purple), cone photoreceptors (green), and amacrine cells (yellow line). Late progenitors generate rod photoreceptors (magenta line) from E12/GW6, bipolar cells (red line) from E16/GW14 and Müller glial cells (light blue) from E18/GW18 until P7/GW30.

In order to generate all retinal cell types, RPCs first undergo repeated cell divisions to increase the proliferating cell pool before chronologically leaving the cell cycle. Thus, some RPCs leave the cell cycle earlier to originate early cell types (ganglion and horizontal cells), while others stay in the cell cycle to generate late cell types (bipolar cells and Müller's glia) (Martins and Pearson, 2008). As mentioned previously, the cell generation sequence is well-conserved in vertebrate species but the specific day that each cell type exits the cell cycle will differ (Table 1, Figure 2). For ganglion cells, the generation period occurs from E11 to E19 in mouse (Dräger, 1985), and from gestational week (GW) 6 to GW14 in the human fovea (finishing at GW30 in the whole retina).

**Table 1:** Comparison of the temporal course of retinal development in mice and in humans.

**Developmental milestone**	**Mouse**	**Human**
Formation of the optic cup	E9–9.5 (Heavner and Pevny, 2012)	GW5 (Smirnov and Puchkov, 2004)
Pigmented epithelium layer separated from the visual layer	From E13 (Fan et al., 2016)	From GW6 (Smirnov and Puchkov, 2004)
Ganglion cells generation period	E11–E19 (HRP retrograde labeled cells and ^3^H-Thy; Dräger, 1985) E8–E16 (peak E12) (Brn3a labeled cells and BrdU; Voinescu et al., 2009)	GW6 to GW14 in the fovea (finishing at GW30 in the whole retina)
IPL appearance	E17 (Fan et al., 2016)	GW8-9 in the fovea; GW15 temporal and GW18 far periphery (Hendrickson, 2016)
OPL formation	P4–P5 (Olney, 1968)	GW11 (fovea); GW30 (far periphery) (Hendrickson, 2016; Hendrickson and Zhang, 2017)
Ganglion cell PCD	Peak P2–P4 (Young, 1984) and P15 (Péquignot et al., 2003)	Peak GW15–20 (Georges et al., 1999)
PCD in the INL	Inner INL P0–P11 (peak P4–P6) Outer INL P5–P18 (peak P8–P10) (Young, 1984)	From GW15–35 (peak GW20) mainly in the bipolar location
PCD in the ONL	Inner rods P5–P11 (peak P7–8) Outer rods P5–P21/24 (peak P15, Young, 1984; Péquignot et al., 2003)	Significantly lower than other layers (GW15-GW35)
Synapses in the IPL	Conventional synapses P5 (Fisher, 1979) Ribbon synapses P10–P12 (Olney, 1968; Fisher, 1979)	GW12 (future fovea) (Hollenberg and Spira, 1973)
Synapses in the OPL	P7 (Olney, 1968)	GW12 in the future fovea (Hollenberg and Spira, 1973)
Invasion of vascular cells from optic disc	From P2 (Young, 84)	From GW14-15 (Hughes et al., 2000)

*E, Embryonic day; P, Post-natal day; PCD, Programmed Cell Death; GW, Gestational week*.

After the cell cycle exit, immature neuron/glial cells will migrate to a portion of the tissue, forming the retinal layers. According to the fact that ganglion cells are the first cell type to exit the cell cycle, the ganglion cell layer is the first to appear. These cells begin to differentiate soon after their generation and begin neurite growth, forming the inner plexiform layer (IPL). RPCs maintain the generation of new cells which migrate and form other retinal layers in a chronologically maintained sequence of INL, ONL, OPL. In humans, Smirnov and Puchkov (2004) described the migration of RPCs, forming INL and ONL from GW7, which maintains a proliferative state at these layers. The generated cells began to differentiate and generate synapses. The first synapses in the IPL and OPL were visualized at GW12 in the future fovea of human retina (Hollenberg and Spira, 1973), whereas synaptophysin, a good marker for synaptogenesis, only appeared in the OPL at GW16 (Nag and Wadhwa, 2001). In mouse retina, a small but already detectable number of conventional synapses in the IPL first appear at P5 (Fisher, 1979) and the first few ribbon synapses in the IPL, from P10-P12 (Olney, 1968; Fisher, 1979). In the OPL, ribbons in photoreceptors could be seen from P2, whereas synaptic terminals with synaptic vesicles making synaptic contact with other cells are only visualized from P7 (Olney, 1968).

Retinal development, as well as in the brain, involves programmed cell death (PCD) which plays a very important role in tissue refinement, regulating the number of specific cell types leading to a mature tissue. PCD follows a central-peripheral gradient, similarly to the other retinal development events (Chavarría et al., 2013). Four PCD phases are recognized in mouse retinal development: (1) morphogenic cell death associated to cell death of the optic cup invagination and closure of the optic fissure; (2) early neural cell death targeting proliferating neuroepithelial cells and recently born neurons and glia; (3) neurotrophic cell death affecting differentiated neurons competing for neurotrophic supply and regulated by activity-dependent processes, associated with intra- and extra-retinal synaptogenesis; and (4) a late phase, with a peak at P15, probably related with intraretinal synaptogenesis (Laemle et al., 1999; Péquignot et al., 2003; Vecino et al., 2004; Valenciano et al., 2009; Chavarría et al., 2013; Braunger et al., 2014; Francisco-Morcillo et al., 2014; Vecino and Acera, 2015).

Morphogenic PCD is higher at E10.5, with most of apoptotic cells located in the middle of the retina, decreasing progressively from E11.5 to E13.5 (Péquignot et al., 2003; Valenciano et al., 2009), when fissure closure in mice ends, by E13 (Strongin and Guillery, 1981). Early PCD was described from E15.5-E17.5 (Péquignot et al., 2003) but recently it has been reported that the elimination of RGCs also occurs from E12.5 to E16.5, through microglia phagocytosis (see below; Anderson et al., 2019). Neurotrophic PCD consists of two waves, the first initiating at E18.5, when cell death begins to increase in the inner neuroblastic layer and peaks at P2-P4, and the second peaking at P9 (Young, 1984; Péquignot et al., 2003). At P0, neurotropic PCD occurs at a higher rate in the GCL, with a peak at P2-P4 (Young, 1984). Georges et al. (1999) evaluated PCD in human retinas from GW15 to GW35 and found apoptotic cells in all layers during this period. However, the highest rate of cell death at GW15 was found in the GCL, which was greatly decreased by GW23-24 (Georges et al., 1999). The cell death of ganglion cell population leads to a substantial loss of axons in the NFL (~70%) from GW15 to GW30 (Provis et al., 1985). In the INL, the incidence of apoptotic cells was two to eight times that observed in the GCL, peaking at GW20 (Georges et al., 1999). Interestingly, 85–90% of the apoptotic cells in the INL occupied the middle and outer third location, suggesting that most INL cells undergoing PCD were bipolar cells. Young (1984) observed the same phenomenon in the INL of mouse retina, with significantly higher incidence of pyknotic cells in the bipolar/Müller cell position. The window of cell death in the inner/outer INL was also slightly different, with inner INL undergoing PCD from P0 to P11 (peak P4–P6) and the outer INL from P5 to P18 (peak P8–P10) (Young, 1984). PCD of cells in the ONL remain low throughout retinal development, and occurs later on from P5 to P11 (peak P7–P8) for the inner rods (differentiating rods found in the inner part of the ONL) and, at a significantly lower rate, for outer rods in the ONL from P5 to P21/24 (Young, 1984). Péquignot et al. (2003) reported a peak at P15 in ONL as well as a second peak in the GCL. PCD in the ONL of the human retina also occurs at a significantly lower rate than other layers with few pyknotic nuclei found from GW15 to GW35 (peak at GW23–GW24) (Georges et al., 1999).

The mechanisms of these PCD phases in the mouse retina can be distinctly regulated. Morphogenic PCD related to optic fissure closure involves BMP, FasL and Msx2 (Péquignot et al., 2003; Wu et al., 2003; Francisco-Morcillo et al., 2006), whereas neurotrophic PCD involves apoptotic signals, such as caspases and Bax (for a review, see Valenciano et al., 2009). In the mouse retina, up to 60% of recently born retinal ganglion cells die after birth in a Bcl2 dependent way (Bonfanti et al., 1996; Strettoi and Volpini, 2002; Péquignot et al., 2003). Furthermore, BDNF/trkb controls the dynamic of RGC death after birth (Pollock et al., 2003). Trafficking of apoptotic signals through gap junctions also regulates PCD in INL and GCL (Cusato et al., 2003). There is also a suggestion of caspase-independent cell death mediated by PARP-1 and AIF nuclear translocation during the first postnatal week (Marín-Teva et al., 2011). Several other studies on different species, have demonstrated the important role of neurotransmitters and signaling pathways, such as glutamate, ATP, insulin, integrins, cyclic AMP and nitric oxide in PCD during retinal development (Martins et al., 2005; Valenciano et al., 2009; Cossenza et al., 2014; Ventura et al., 2019a).

In the developing retina, dying cells are phagocyted mainly by microglia, but also by neuroepithelial cells and Müller cells, enabling a clean removal of dead cells (Francisco-Morcillo et al., 2014; Silverman and Wong, 2018). In chick embryo retina (E4), engulfment and lysosomal degradation of apoptotic bodies seem to depend on autophagic cell death (Mellén et al., 2008). Microglia are already present at E11.5, shortly after the onset of retinal neurogenesis (Santos et al., 2008). At E12.5 and E14.5, microglia primarily associate with neurons, especially ganglion cells (Anderson et al., 2019). During this period, a small percentage of microglia contact cleaved-caspase 3-positive cells (15% at E12.5 and 7% at E16.5) (Anderson et al., 2019). Anderson et al. have shown that microglia phagocyte non-apoptotic neurons, in a process called phagoptosis, regulating the elimination of RGCs in the early stage of PCD (Anderson et al., 2019). The PCD of these neurons is also stimulated by Nerve Growth Factor through p75 receptor, in early (E13-15.5) but not later (E17) stages (Frade and Barde, 1999; Harada et al., 2006).

## Congenital Torch Diseases

The acronym TORCH is globally used to encompass pathogens known for their teratogenic effects, namely ***T****oxoplasma gondii*, ***R****ubella virus*, ***C****ytomegalovirus*, and ***H****erpes virus*. Currently, **O** stands for ***O****thers* and can include syphilis, parvovirus, coxsackievirus, listeriosis, hepatitis virus, varicella-zoster virus, *Trypanosoma cruzi*, enterovirus and human immunodeficiency virus (HIV). Recently, the Zika virus was also included in this list, after the 2015–2016 outbreaks in Latin America, which were correlated with a high number of microcephaly cases (Schwartz, 2017). All these diseases are teratogenic, i.e., can cause disturbances in fetal development, leading to malformations. Neurotropism also occurs among the aforementioned microorganisms. In this topic, we will present the main teratogenic pathogens, their characteristics, and how they affect development, focusing on eye abnormalities, with special focus given to Congenital toxoplasmosis.

The placenta is the first biological barrier that TORCH pathogens must overcome in order to reach fetal tissues. During normal placental development, invasive cytotrophoblasts (CTBs) originating from anchoring chorionic villi invade the maternal decidua. CTBs are specialized epithelial cells of the placenta which leave the basement membrane and differentiate along two independent pathways, depending on their location, to initiate the blood flow to the placenta. A subset of these cells remodels the uterine vasculature in the decidua at the maternal-fetal interface. This process is finely controlled through the coordinated actions of invasion- and angiogenesis-promoting factors. The maternal decidua exhibits a distinctive multicell nature, comprising invasive CTBs and uterine epithelial, stromal, and endothelial cells, as well as immune cells (reviewed by Maltepe et al., 2010).

Trophoblast cells of the human placenta, derived from the outer cell mass of the blastocyst, are at the center of the balance between infection responses and conception tolerance (Heerema-McKenney, 2018). Vertical transmission (mother to fetus) of pathogens can occur by several routes, including the following: infection of endothelial cells in the maternal microvasculature and spread to invasive extravillous trophoblasts, which anchor the villous trees to the uterine wall, trafficking of infected maternal immune cells across the placental barrier, paracellular or transcellular transport from the maternal blood across the villous trees and into the fetal capillaries, damage to the villous tree and breaks in the syncytiotrophoblasts layer, and/or transvaginal ascending infection (Coyne and Lazear, 2016). Most TORCH agents are thought to infect the placenta and fetus from a hematogenous route, although infection from cervical shedding or decidua infection may also occur.

A recent study using single-cell RNA-Seq has demonstrated that placental cells express NRP2, PDFGRA and CD46 receptors, which permit CMV invasion to host cells (Pique-Regi et al., 2020). CMV may cross the placenta via transcytosis of first-trimester syncytiotrophoblast cells and, in an *ex vivo* infection decidual organ culture model, HCMV infects invasive cytotrophoblasts, macrophages, and endothelial, decidual and dendritic cells (Weisblum et al., 2011). ZikV has been shown to infect syncytiotrophoblasts, cytotrophoblasts, decidual, and endothelial cells, leading to increased inflammation response, including CD68 and CD8 cell infiltration and cytokines, chemokines and MMP secretion (Rabelo et al., 2020). Additionally, placental cells at birth (mean gestational age 36 weeks) were shown to express AXL, CD209 and TYRO3, which may serve as preferential receptors for the Zika virus entry (Pique-Regi et al., 2020). Specifically, AXL was found to be expressed in placenta cells and chorioamniotic membranes, whereas CD209 was mostly expressed in maternal and fetal macrophages subsets. In the same study, C1QBP (Complement component 1 Q subcomponent-binding protein) and CALM1, both known Rubella virus interactors (Mohan et al., 2002; Zhou et al., 2010), were expressed in syncytiotrophoblasts throughout the pregnancy, and to a lower extent in decidual, endometrial and cytotrophoblast cells (Pique-Regi et al., 2020). Regarding congenital toxoplasmosis, the *in vivo* mechanisms of human transmission are poorly understood. Using *in vitro* explants of human first trimester villous, Robbins et al. (2012) demonstrated that extravillous trophoblast of anchoring villi are most susceptible to infection, followed by villous cytotrophoblast and rare *foci* of syncytiotrophoblast infection observed near damage areas. These findings suggested that maternal parasitemia likely leads to decidual tissue seeding, with subsequent spread to extravillous and villous cytotrophoblast through anchoring villi (Robbins et al., 2012). Histopathological examinations have shown that the placenta may exhibit lymphohistiocytic chronic villitis, with severe and diffuse inflammation and granulomas, immature villi and increased Hofbauer cells in the villous stroma, chorion, and Wharton jelly (reviewed by Costa et al., 2020). Although, the teratogenic effects of each TORCH agent is probably caused by different mechanisms, placental inflammation is possibly an important player in a CNS development context, by increasing cytokine production from reactive microglia and astrocytes and altering neurotransmitters expression/activity (al-Haddad et al., 2019).

### Congenital Rubella Syndrome

*Rubella* is a common disease whose etiological agent is the *Rubella* virus (RV). Belonging to the *Togaviridae* family, this single-stranded RNA virus is transmitted by direct contact or by droplets through respiratory secretions. It is of extreme concern when infecting pregnant women, due to its teratogenic ability (Frey, 1994). The rate of congenital infection following maternal rubella has been reported as 85% in the first trimester, 54% at 13 to 16 weeks, 36% at 17 to 22 weeks, 30% at 23 to 30 weeks, and then 60% at 31 to 36 weeks, with an impressive 100% transmission rate in the last month of pregnancy (Freij et al., 1988; Boppana et al., 2017). In addition to causing miscarriages, congenital rubella syndrome is a major cause of blindness, deafness, heart disease and intellectual disability. These clinical manifestations and the ability of RV to cross the placenta, causing development impairment, are similar to those of other TORCH pathogens (Robertson et al., 2003).

Ophthalmic pathologies are commonly found in congenital rubella. Cataract was the first reported teratogenic effect of gestational rubella (Gregg, 1941), as well as retinal defects, iris adherence to the lens, microphthalmia (Töndury and Smith, 1966), subretinal vascularization and glaucoma (Freij et al., 1988). Pigmentary retinopathy and strabismus are additional examples of abnormalities in this condition. Each clinical manifestation mentioned above is closely correlated with the gestational period in which the primary infection occurs (Duszak, 2009), with the first trimester exhibiting the most damage (Boppana et al., 2017). Viral particles were found in the ciliary body and lacrimal glands, which can contribute to cataractogenesis (Nguyen et al., 2015). Studies have shown that RV infection causes changes in actin filaments, which appear as amorphous clusters, presumably due to actin depolymerization (Bowden et al., 1987). This lack of organization of actin cable/bundles can result in cell division inhibition. In fact, decreased mitotic activity has been demonstrated in infected primary embryonic cell cultures, while cell division deceleration has been reported in human fetal cells infected by RV (Rawls and Melnick, 1966; Bowden et al., 1987). The rubella virus non-structural protein, P90, can interact with important cell cycle regulators, retinoblastoma and cytokinesis-regulatory proteins, thus influencing cell cycle and apoptosis (Atreya et al., 2004; for a review, see George et al., 2019). Furthermore, downregulation of genes involved in sensory organs and eye development have also been reported by gene expression profiling of RV-infected human umbilical vein endothelial cells (HUVEC) (Geyer et al., 2016). RV infection can induce apoptosis in several cell types involving classic signaling pathways, leading to the activation of caspases, p53, p21, and Bcl-2 family proteins. However, PCD induced by RV infection is observed only in non-proliferative and differentiated cells (for a review, see George et al., 2019). These data suggest that apoptosis is not involved in the regulation of mitotic rate of progenitor infected cell populations. These alterations may explain why RV infection is associated with loss of eyesight as an ophthalmic sequelae (Geyer et al., 2016). Current studies on congenital rubella indicate that such ophthalmic sequelae may be correlated to the regulation of genes involved in the development of sensory organs and to changes in the host cell cytoskeleton that may lead to changes in mitotic pattern (George et al., 2019). However, the molecular/cellular mechanisms responsible for multiple retinal defects in CRS are still poorly understood. Few studies investigating this topic are available, most focusing on histopathological analyses performed during the autopsy of aborted or dead fetuses.

### Congenital Cytomegalovirus Infection

*Cytomegalovirus* (CMV) is a worldwide widespread member of the *Herpes virus* family. In healthy people it is asymptomatic, thus characterizes as an opportunistic microorganism (Landolfo et al., 2003). Affecting about 60% of the population in developed countries, and reaching 100% in developing countries, CMV behaves similarly to the *Herpes simplex virus* (HSV). After primary infection (symptomatic or not), the virus goes into latency and reactivation can occur in situations of low immunity (Griffiths et al., 2015). Cytomegalovirus can be transmitted through direct or indirect contact with infectious body fluids like saliva, urine, blood, semen, or cervical or vaginal secretions. Maternal CMV infection is mainly acquired through contact with the urine or saliva of infected individuals or sexual contact (Cannon, 2009). Like the other pathogens described in this review, CMV can cross the transplacental barrier, and is one of the most hazardous TORCH pathogens.

Congenital CMV infection is common in humans, since maternal immunity is unable to prevent reactivation of the virus during pregnancy and prevent transmission to the fetus (Alford et al., 1980). Factors influencing fetal transmission rates are the trimester of exposure, maternal age, CMV serological status, maternal immunological status and viral load (Ghekiere et al., 2012). Congenital CMV infection can occur even if the infection occurred before pregnancy (non-primary infection). Forms of transmission to the fetus and baby include the transplacentary route, perinatal route (during delivery) by cervical secretions and blood or by breastfeeding (Malm and Engman, 2007).

The main clinical manifestations of congenital infection commonly found in neonates are thrombocytopenia, jaundice, hepatosplenomegaly, microcephaly and retinochoroiditis (Bale, 2014). Some ophthalmic changes caused by congenital CMV infection may be observed in symptomatic and asymptomatic patients. Among those found in both cases are macular scars, strabismus, retinochoroiditis and anterior stromal corneal scars. Symptoms found only in symptomatic patients include peripheral retinal scars, optic atrophy, optic nerve hypoplasia, coloboma, microphthalmia, anophthalmia, and incomplete cyclopia (Ghekiere et al., 2012).

Although congenital defects caused by CMV infection are well-recognized, their pathogenesis is still poorly understood. This is due to the fact that it is difficult to establish adequate animal models for this type of study, since the virus exhibits infectivity in a species-dependent manner. Some studies focused on understanding the effects of CMV infection on neurodevelopment and providing a basis for understanding the damage caused to fetuses have been carried out (Cheeran et al., 2009; Kawasaki et al., 2017). It has been demonstrated in a murine model that developmental damage may be associated to the type of embryonic cells susceptible to CMV infection and to the effects of the infection on their cellular functions. Mesenchymal stem cells are infection targets during mid-pregnancy, affecting brain, eye and orofacial region organogenesis (Tsutsui et al., 1993). Using neural precursor cell neurospheres obtained from the forebrain of aborted human fetuses during the first trimester as an *in vitro* model, it was demonstrated that HCMV inhibits neuronal differentiation induction and provokes apoptosis in infected cells (Odeberg et al., 2006). More recently, studies using cerebral organoids derived from human-induced pluripotent stem cells have indicated that CMV infection can lead to severe damage to the organoid structure, in addition to resulting in calcium signaling and neural network activity alterations. The infection dramatically affects organoid neurological development, reaching the developing cortical structure to fully formed ones, with associated changes in architecture organization and lamination depth within these structures. Such changes may be correlated with microcephaly in human fetuses (Brown et al., 2019; Sun et al., 2020).

### Congenital Herpes Simplex Infection

The *Herpes simplex virus* (HSV), a member of the family *Herpesviridae* viruses, has an enveloped DNA capable of multiplying in the host cell nucleus (Liesegang, 2001). HSV transmission is dependent on mucosal or injured skin contact between a susceptible seronegative individual and another who excretes HSV. Two herpes simplex virus serotypes are known, HSV-1, correlated with oral lesions, and HSV-2, associated with genital lesions. Both viral serotypes establish latent infection in sensory neurons and, when reactivated, cause lesions near or at the body's entry sites (James and Kimberlin, 2015). Approximately 5% of neonatal HSV infections occur *in utero*, 85% during the peripartum period, and the remaining 10%, postnatally, through direct contact with infectious lesions or secretions (Brown et al., 1997).

Congenital HSV infection is associated with high levels of morbidity and mortality. The most common form of transmission occurs at birth, through direct contact with lesions or by asymptomatic viral shedding (Fa et al., 2020). Transplacental HSV transmission was first reported in 1963, in which the newborn exhibited herpetic lesions at birth. During developmental follow-up, several neurological damages associated with congenital HSV infection were observed, such as strabismus, retinochoroiditis, hyperreflexia, and slow speech development (Mitchell and Mccall, 1963). Additional ocular abnormalities such as chorioretinitis, microphthalmia, keratoconjunctivitis and optic atrophy are also found in congenitally infected individuals (Leung et al., 2020).

Although vertical transmission by HSV is considered rare, like other TORCH pathogens, the greatest risk of infection occurs during early pregnancy (Fa et al., 2020). *In vitro* HSV infection models of neural progenitor cells may give clues that may aid in understanding what occurs in the developing CNS, including the brain. It has been demonstrated *in vitro* that HSV can infect undifferentiated iPS cells, neural precursors cells and iPS-derived differentiated sensory neurons (Lee et al., 2012). Infection by HSV is highly cytotoxic to neural progenitor cells, unlike infection by the Varicella Zoster virus (VZV), which does not infect undifferentiated iPS cells. Similarly, HSV-1 can successfully infect human embryonic stem cells, whereas VZV does not (Dukhovny et al., 2012). In the adult mouse brain, ependymal and neural stem cells express the Herpes virus entry mediator protein (HVEM) and *in vitro* studies concerning the infection of such cells indicate reduced neuronal generation rates, as shown by doublecortin (DCX) immunostaining, which was prevented by microglia-derived IL-6 secretion (Chucair-Elliott et al., 2014). Infection of mouse neural stem cells by HSV in a neurosphere model leads to cell death, with reduction in neurosphere size and the production of IFN-γ mediated by Toll-like receptor 3 activation (Sun et al., 2015). HSV-1 also activates the JNK and p38 MAP kinase signaling pathways, which further contribute to cytolytic host cell effects (Zachos et al., 1999; Diao et al., 2005; Hargett et al., 2005). In turn, p38 and JNK are known apoptosis regulators and may be implicated in neurodegeneration and brain and retina neurogenic defects (Shou et al., 2003; Diao et al., 2005; Dhanasekaran and Reddy, 2008; Shklover et al., 2015; Wang et al., 2018; Kawamura and Kano, 2019; Kovacs et al., 2019; Lei et al., 2020; Pang et al., 2020). Although no direct evidence has indicated direct effects of HSV infection to retinal progenitor cells, either *in vitro, in vivo* or in human cases, it is tempting to assume that, similarly to what is observed in cortical progenitor cells, retinal progenitor cells may be susceptible to HSV infection, resulting in similar neurogenesis and apoptosis effects.

### Congenital Zika Virus Infection

The *Zika virus* (ZikV) is an arbovirus, displaying a classic human-arthropod-human vector transmission pathway. Belonging to the *Flaviviridae* family, it was first described in 1947 (Dick, 1952). Initially, the pathogenesis of this disease was considered mild, characterized by fever, rash, joint pain and conjunctivitis. However, the relevance of ZikV infection increased after an outbreak in 2015 in northeastern Brazil, where a sudden increase in the birth of neonates with microcephaly was observed (Rasmussen et al., 2016). This increase was correlated with primary maternal ZikV infection during pregnancy after confirmation of viral genetic material in the amniotic fluid of pregnant women with microcephalic fetuses (Calvet et al., 2016; de Araújo et al., 2016). Thus, it has become clear that in addition to classical transmission, ZikV is transmitted sexually and congenitally and is highly teratogenic (de Araújo et al., 2016). Therefore, ZiKV is considered an important member of the TORCH pathogen group (Musso and Gubler, 2016). Some authors suggest modifying the old acronym TORCH for new TORZiCH to highlight the position of Zika virus due to the serious congenital disorders associated with ZikV infection (Tahotná et al., 2018).

The emerging association of congenital ZikV infection with microcephaly demanded the beginning of research in the area to identify possible damage to the offspring and preventive or curative interventions. Among animal models, the mouse model has been widely applied in several ZikV infection studies (Caine et al., 2018). Among the reported damage from congenital pathogenesis caused by ZikV are primary microcephaly and microphthalmia. Although infection of neural progenitors, neurons and glial cells have been described (Cugola et al., 2016; Gabriel et al., 2017; Büttner et al., 2019; Ferraris et al., 2019), a recent study based on *in vitro* research suggests that the primary targets of ZikV are astrocyte cells (Ledur et al., 2020). Such infection also affects cell migration, neurogenesis, differentiation and cell death, leading to microcephaly in neonates (Russo et al., 2017; Wen et al., 2017; Christian et al., 2019).

Eye abnormalities and visual problems are also observed in neonates congenitally infected with ZikV (Ventura et al., 2019b; Lima et al., 2020). Clinical manifestations include microphthalmia, retinal pigment changes, chorioretinal atrophy, vascular changes and optic nerve hypoplasia (Ventura et al., 2016). Such anomalies opened a precedent for studies on the development of the infected offspring retina. ZikV infection in pregnant mice generated decreased eyeballs, optic nerve thinning, retinal damage and impaired visual projection (Shi et al., 2018) and impaired vascular offspring development (Garcez et al., 2018).

It is still unclear which are the cellular targets of ZikV in the developing human fetus. *In vitro* studies have reported that ZikV infects human embryonic cortical neural progenitor cells (hNPCs), inducing cell cycle dysregulation and increased cell death (Tang et al., 2016). In addition, by studying the mechanisms by which ZikV modulates the cell cycle of hNPCs, it has been observed that the virus induces DNA breaks which, in turn, inhibits cell cycle progression from the S phase, thus preventing host DNA replication completion (Hammack et al., 2019). Using an *in vivo* congenital ZikV infection model, it has been verified that, besides neurogenesis impacts, the infection also affects angiogenesis. When compared to control offspring, ZikV-infected offspring exhibited decreased blood vessels in the vasculature of both the cerebral cortex and the retina (Garcez et al., 2018). Using intrauterine infection as a vertical transmission model, congenital Zika syndrome has been shown to generate mice with smaller eyeballs and smaller optic nerves. Additionally, a reduction in the thickness of GCL, IPL, and ONL and the absence of OPL was also detected, which could be correlated to visual neural connection defects. ZikV infection also decreased the number of ganglion cells in the GCL, which is clearly associated to optic nerve damage (Shi et al., 2018). It is known that retinal endothelial cells, retinal pericytes and retinal pigmented epithelial cells are permissive for lytic ZIKV replication and primary retinal barrier target cells concerning infection (Alcendor, 2019). These data can contribute to elucidate how ZikV affects retina development and the mechanisms involved in the pathogenesis of retina lesions after congenital infection.

### Congenital Toxoplasmosis

Toxoplasmosis is a zoonosis of great interest in the context of public health, since it affects a third of the world population, with the protozoan parasite *Toxoplasma gondii* as etiological agent (Tenter et al., 2000). Among the TORCH agents, *T. gondii* is the main protozoan representative, while most display a viral etiology. Toxoplasmosis is widely distributed across the countries, reaching seropositivity rates that vary from <10% to over 90% (Torgerson and Mastroiacovo, 2013). Among the infected population, two groups of medical importance are highlighted, immunocompromised persons and those congenitally infected, in which the most severe forms of the disease are observed (Furtado et al., 2011).

In Brazil, seroprevalence reaches very high numbers, of over 60% (Ozgonul and Besirli, 2016), with the presence of anti-*T. gondii* antibodies present in up to 50% of children in primary school and 50–80% of women in fertile age. The rates of congenitally infected children in Brazil are also high, reaching 5–23 born infected out of 10,000 born alive in Brazil (for review, Dubey et al., 2012). Some factors are determinant for such a high seroprevalence, such as scholarity and low family income. Therefore, social vulnerability may play an important role in high seroprevalence rates (Mareze et al., 2019). Toxoplasmosis is still relevant in Brazil and frequent outbreaks are observed, such as the one that occurred in 2018 in the city of Santa Maria, in which 1,116 cases were reported by public health agents, with another 766 suspect cases (Dal Ponte et al., 2019).

*Toxoplasma gondii* is an opportunistic protozoan that belongs to the Apicomplexa phylum, first described by Nicolle and Manceaux (1908) in rodents in North Africa (Ferguson, 2009). In that same year, it was described in Brazil in the state of São Paulo by Splendore, thus suggesting that *T. gondii* is a cosmopolitan parasite (Frenkel, 1973).

*T. gondii* is an intracellular obligate parasite capable of infecting virtually all nucleated cells in the host, thus reaching different tissues, with a preference in forming tissue cysts in muscle and neuronal cells (Dubey, 2004). The ability of the parasite to infect cells and persist in the tissues in a latency state, i.e., tissue cysts, contribute to toxoplasmic retinochoroiditis. Chronic infection reactivation by *T. gondii*, and consequently, the disease, is common in congenitally infected individuals. Different mechanisms are pointed as responsible for recurrent ocular toxoplasmosis (OT): first, the destruction of retinal tissue may be due to the release of parasites from tissue cysts, which in turn invade and promote the lysis of adjacent cells; second, the immune response generated against this parasite may be harmful for the host's tissue (Roberts and McLeod, 1999). The classic clinical aspect observed in patients is an active lesion and a fresh white elevated focus of a necrotizing lesion, proximal to a previous pigmented scar (Pavesio and Lightman, 1996). Retinochoroiditis can be incapacitant, with most cases observed in young adults, correlated to untreated congenital toxoplasmosis. Because of this, CT has great medical and socio-economic relevance, and is the reason for the creation of pre- and neonatal triage programs (Wong, 2006).

The first reported case of infantile toxoplasmosis with confirmed vertical transmission dates back to 1942 (Cowen et al., 1942). Currently, it is known that the congenital infection is the most severe form of toxoplasmosis and occurs in the offspring of mothers that contracted primary *T. gondii* infection during pregnancy. The diagnosis is made through serological testing for *T. gondii* or based on abnormal ultrasonography examination (Bollani et al., 1967). Around 90% of individuals that acquire toxoplasmosis will not exhibit typical signs and symptoms, which makes the diagnostic even more difficult. In addition, symptoms (fever, nausea and lymphadenopathy) are easily mistaken with those of other non-teratogenic infections (Hampton, 2015).

The incidence and severity of congenital toxoplasmosis infection depend on the gestational period when infection occurs. The risk of vertical transmission increases over the gestational weeks, of 15% in the 13th week, 44% in the 26th and 71% in the 36th, increasing to 90% in the last week of pregnancy. However, severity of the damage to the fetus is inversely proportional to the infection period. Severe manifestations are seen in the offspring of women who acquired the infection during the beginning of the pregnancy whereas it may be subclinical in neonates born to mothers infected at the end of the pregnancy (Hall, 1992). Placenta physiology plays an important role and is closely related to infectivity rates, since it is immunologically responsible for avoiding maternal-fetal rejection and for preventing vertical infection (Wong, 2006).

CT may manifest in the first month of life or be noticeable as long-term ocular and neurological sequela in childhood or even adulthood. The main consequences include spontaneous abortions, neurological disturbances and ocular damage (Randall and Hunter, 2011). Ophthalmological manifestations are among the main sequelae in CT, and retinochoroiditis is the most common, with an estimated incidence of 9–31%. Other possible ocular manifestations include strabismus, microphthalmia, cataracts, retinal detachment, optic atrophy, iridocyclitis, nystagmus and glaucoma. Some of these characteristics are apparently correlated with a retinochoroiditis process that is then applied as a marker of CT severity (Bollani et al., 1967).

Despite ocular lesions being frequently correlated with CT, they can be found after infection even in immunocompetent hosts (Gazzinelli et al., 1994). For example, retinal neovascularization, a rare complication of ocular toxoplasmosis (OT) can be a source of vitreous hemorrhage (Gaynon et al., 1984). Other less common abnormalities may be noticeable, such as vascular occlusion even far from active lesions, thus resulting in hemorrhage. Retinal detachment and subretinal neovascularization have also been reported (Nussenblatt and Belfort, 1994).

The first description of OT was made by Jankû in 1923, followed by Levaditi in 1928. However, the relationship between *T. gondii* infection and retinochoroiditis was only reported in 1952, as reviewed by Kim and Weiss (2004). OT has been widely studied since then, although it still poses many challenges regarding its physiopathology (Maenz et al., 2014). In OT, the first tissue to be affected is the retina, followed by the choroid, the vitreous humor and anterior chamber, which can all be affected, but never before the retina (Nasaré and Tedesco, 2017). Uveitis and retinochoroiditis are clinical aspects characterized by the inflammation of the uveal tract that can occur during the pathogenesis of OT, possibly evolving to irreversible ocular lesions (Holland, 1999).

Although not completely clear, it is thought that *T. gondii* reaches the retinal tissue using a Trojan horse mechanism, being transported by an infected inflammatory cell through the Blood-Retinal Barrier, similarly to described in brain invasion (Kijlstra and Petersen, 2014; Lachenmaier et al., 2014). In experimental models, *T. gondii*-infected THP-1 monocytes have been reported as transmigrating monolayers of human retinal pigmented epithelial cells (ARPE-19) (Song et al., 2017), whereas direct infection of ARPE-19 cells affects their junctional properties, including decreases in Transepithelial (/endothelial) Electrical Resistance (TEER) (Nogueira et al., 2016). Regarding the Blood-Retinal Barrier, Furtado and colleagues have indicated that *T. gondii* can cross through retinal endothelial cells without disturbing the integrity of the monolayer (Furtado et al., 2012), thus penetrating the retinal layers and infecting neuronal and glial cells (Furtado et al., 2013). Similarly, *T. gondii* has been shown to infect cerebral microvascular endothelial cells, which may serve as a niche to gain entry to the brain (Konradt et al., 2016).

It is noteworthy that *T. gondii* utilizes an intricate mechanism to disseminate through the host organism, traveling in infected inflammatory cells, including dendritic cells, monocytes and lymphocytes, that acquire a hypermigratory phenotype (Da Gama et al., 2004; Seipel et al., 2010; Fuks et al., 2012; Kanatani et al., 2015, 2017; Ueno et al., 2015; Ólafsson et al., 2018; Bhandage et al., 2019). This phenomenon also holds true for retinal tissue, since infected dendritic cells have been shown to transmigrate across retinal vascular endothelium through adhesion molecules (ICAM-1, V-CAM and ALCAM) and chemokines (CCL21 and CXCL10) (Furtado et al., 2012). Therefore, increasing evidence has shown that *T. gondii* has developed complex mechanisms to penetrate the CNS, either in the brain or retina.

Once inside the tissue, tachyzoites invade host cells and proliferate, leading either to host cell lysis or to the formation of tissue cysts, composed of bradyzoites (Kijlstra and Petersen, 2014). The preference for retinal tissue may be correlated to a higher susceptibility of the vascular endothelium present in the retina to *T. gondii* infection, and such susceptibility may be related to easy penetration in the host cell, intracellular proliferation rates and/or the cellular response to infection (Smith et al., 2004).

Animal infection models used to study OT must recapitulate aspects of the human pathology from the invasion of the host until the development of disease. The first experimental models had the goal of providing a better description of the disease pathogenesis. In order to analyze the migration of the parasite to the retinal tissue, rabbits and hamsters were infected through distinct routes of infection, including the intracarotid and intraperitoneal routes, respectively (Frenkel, 1955). However, such approaches do not mimic the natural route of infection. Throughout the years, an increasing number of studies has focused on experimental modeling of Toxoplasma-induced retinochoroiditis, through the use of non-human primates, cats, rabbits, hamsters and mice. A recent review compared mouse strains, parasite genotypes, disease stage and inoculum dosage, reporting that adult C57bl/6 mice were more susceptible to infection via the oral route and developed OT very rapidly (14 to 21 days post infection, dpi), thus becoming the model to more closely mimic natural infection (Dukaczewska et al., 2015). C57bl/6 mice are more susceptible to infection, with higher lethality rates of mothers and offspring. This makes it more difficult to develop a reliable system to be used as an OT/CT animal model. However, the fact that these animals are pigmented animals makes them a more appropriate model to study retinal biology, as retinal development of albino animals is already impaired. Thus, it is worth reinforcing the importance of prioritizing studies with pigmented animals to increase the chances of clinical translation to humans. Up to this moment, very few studies were conducted in order to directly assess the interaction between *T. gondii* and retinal cell types, neither aiming at characterizing the morphological or functional alterations induced by the parasite, nor looking for specific tropisms for retinal cell types. Lahmar et al. (2010) reported that neonatal infection of Swiss Webster albino mice by *T. gondii* can lead to retinal layer disorganization, especially in the GCL. This same group exhibited a discrete, qualitative alteration in immunoreactivity for vimentin and GFAP in the retinas of infected mice. Moreover, a reduction in the number of cells in the ONL was also observed, thus suggesting photoreceptor depletion (Lahmar et al., 2014). *In vitro* infection of retinal cells obtained from chick embryos or retinal explants from adult or chicks embryos demonstrate that *T. gondii* is capable of replicating in these systems and that this is dependent on polyamine production by the host cells (Moraes et al., 2004). Using RPE and Müller cells, isolated from Lewis rats, Delair et al. (2009) indicated that TNF-α and IFN-γ differentially restrict *in vitro T. gondii* infection, thus indicating that RMC are more susceptible to infection than RPE. Finally, it was recently described that *T. gondii* disrupts correct cytokinesis patterns and the formation of the mitotic spindle in bovine endothelial cells (Velásquez et al., 2019). It is known that changes in spindle structure, with or without cell cycle protein alterations, can lead to abnormal retinal and brain cortex development (Uzquiano et al., 2018), which could also explain how CT affects these processes.

Alterations in the profile of the structures of retinal layers, such as detachment of the pigmented epithelium from the ONL and irregularities in the placement of retinal layers, have been described in the literature in congenitally infected mice, where ocular abnormalities were more evident than in acquired infection (Ashour et al., 2018). However, the literature lacks studies systematically describing whether the damage found in congenital OT is derived from alterations that occur during the proliferation and differentiation of retinal progenitor cells during development.

## Concluding Remarks

Congenital infection by TORCH syndrome agents is a relevant public health threat with varying degrees of severity. In the specific case of the Rubella virus, transmission rates were greatly reduced due to vaccination programs in the 1980s−1990s. However, special attention must be given to antivax movement, which has contributed to increasing the number of Measles-Rubella cases (Hotez, 2019; Krishnendhu and George, 2019). In 2019, 1,241 new measles cases were reported in the United States as a result of this movement (Nathala et al., 2019). Regarding CT, this infection comprises a high epidemiologic burden and its ensuing sequelae are irreversible which, combined with the lack of efficient chemotherapeutic schemes represents an important challenge in terms of basic research that aim to understand the molecular and cellular events that lead to these malformations.

**Figure 3:**
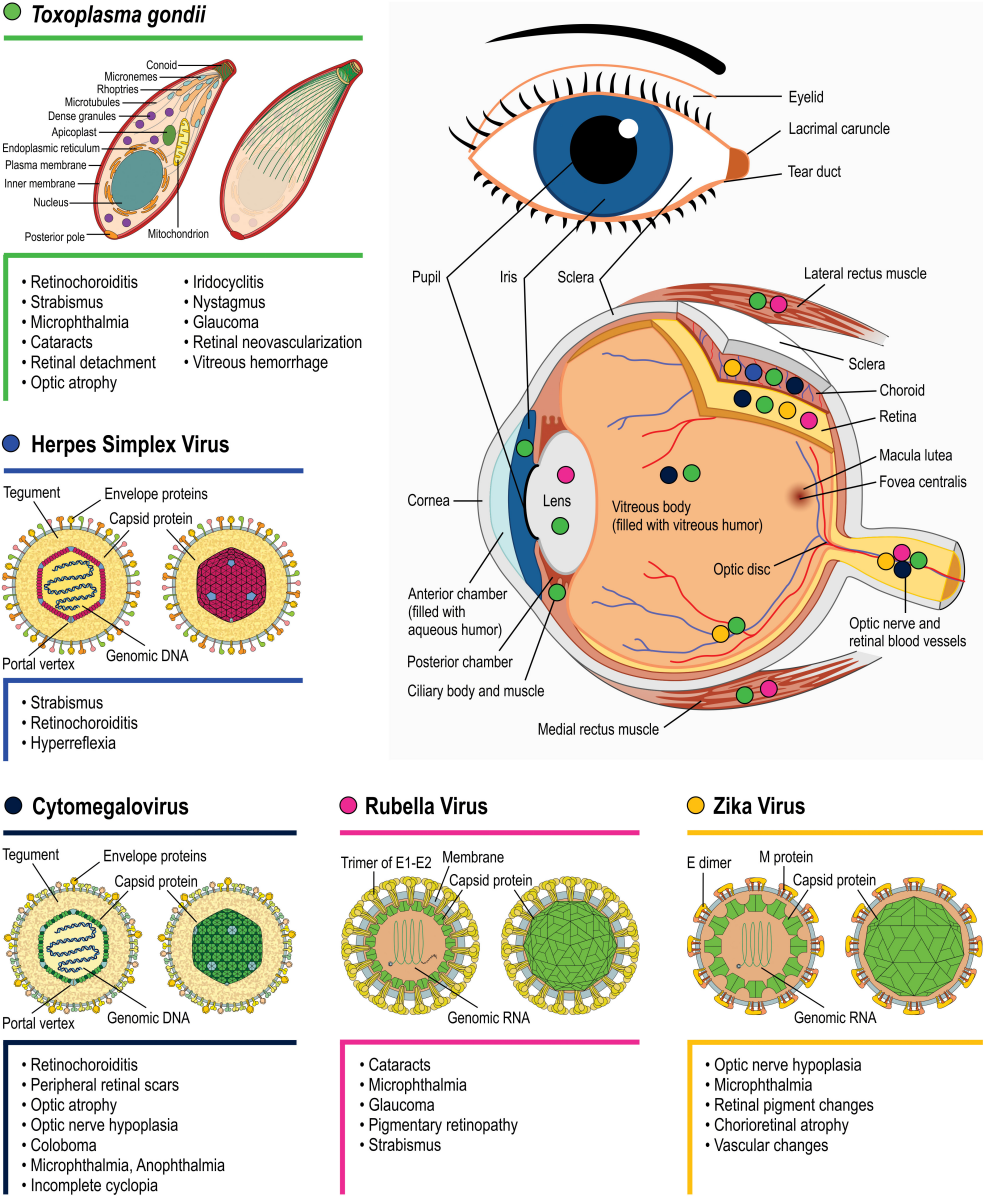
Main eye structures affected by each TORCH agent during development (indicated by colored circles). Clinical consequences regarding congenital *T. gondii* (green symbols), Herpes simplex virus (blue), Rubella virus (magenta), Zika Virus (orange), and Cytomegalovirus (navy blue) infection are listed besides each pathogen's name. Viral structure representations were based on Hulo et al. (2011).

All TORCH infections can cause severe but different neurological disabilities and ophthalmic problems. However, the outcome may differ depending on the pathogen infection, as exhibited in Figure 3. There is still much left to unravel concerning the mechanisms by which each pathogen affects eye/retinal development. One important interfering determinant is the embryonic stage of the infection. As highlighted for each topic, the infection period can vary greatly depending on the pathogen, with 85% of congenital HSV occurring during labor whereas 85% of congenital rubella infection occurs during the first gestation trimester. Evidently, since different retinal development phenomena occur throughout the gestational period, as well as postnatally, different outcomes are expected depending on the infection period. Nevertheless, these differences *per se* are insufficient to explain the distinct retinal lesions observed among TORCH infections. Target host cells tropism and the specific molecular/cellular alterations that each pathogen induces are probably as important as the infection period. In the context of ocular malformations and altered retinogenesis observed during TORCH agent infection, certain questions remain unanswered and may serve as motivation for further research, as follows:

1. What are the consequences of TOR(Zi)CH infection in retinal development? What is the exact impact on cell proliferation, morphological and neurochemical differentiation of different cell types, synaptogenesis, programmed cell death, and vascularization? Given the similarities found between neural and retinal progenitor cells, including their susceptibility to infections and the role of altered mitosis and apoptotic balance, it seems tempting to speculate that these may be common mechanisms by which TOR(Zi)CH agents affect retinogenesis.
2. What cell types in the retina are affected during CT? Does the parasite present a tropism for a specific cell type?
3. Are cases of strabismus and nystagmus related to *T. gondii* infection of lateral and medial rectus muscle tissue?
4. Is there a correlation between *T. gondii* genotype and clinical outcomes?
5. Finally, since some reports demonstrate that SARS-CoV-2 not only is transmitted vertically, but cause disease in the infected newborns, future studies will reveal whether this virus could actually be included as a new TORCH pathogen (Muldoon et al., 2020).

## Author Contributions

VdC wrote the first draft of the manuscript and prepared the figures. KC and DA revised and discussed the manuscript and figures. All authors contributed to the article and approved the submitted version.

## Conflict of Interest

The authors declare that the research was conducted in the absence of any commercial or financial relationships that could be construed as a potential conflict of interest.

## Abbreviations

ALCAM: activated leukocyte cell adhesion molecule
ARPE-19: human retinal pigmented epithelial cells
CCL21: C-C Motif Chemokine Ligand 21
CTB: Cytotrophoblast
CMV: Cytomegalovirus
CNS: Central Nervous System
CRA: Central Retinal Artery
CT: Congenital Toxoplasmosis
CXCL10: C-X-C motif chemokine 10
E: Embryonic day
GCL: Ganglion Cell Layer
GFAP: glial fibrillary acidic protein
GW: Gestational Week
HIV: Human Immunodeficiency Virus
HSV: Herpes simplex virus
HVEM: Herpesvirus entry mediator
ICAM-1: Intercellular Adhesion Molecule 1
IFN-γ: Interferon Gamma
ILM: Inner Limiting Membrane
INL: Inner Nuclear Layer
IPL: Inner Plexiform Layer
iPS: induced pluripotent stem cell
NFL: Nerve Fiber Layer
OLM: Outer Limiting Membrane
ON: Optic Nerve
ONL: Outer Nuclear Layer
OPL: Outer Plexiform Layer
OT: Ocular Toxoplasmosis
P: Postnatal day
PCD: Programmed Cell Death
RGC: Retinal Ganglion Cells
RPC: Retinal Progenitor Cells
RPE: Retinal Pigmented Epithelium
RV: Rubella Virus
TEER: Transepithelial (/endothelial) Electrical Resistance
TNF-α: Tumor Necrosis Factor alpha
TORCH: Toxoplasmosis, Others, Rubella, Cytomegalovirus and Herpes
V-CAM: Vascular cell adhesion protein
ZikV: Zika virus.
